# Cubital Tunnel Syndrome Due to Multiple Intraneural Cysts at Elbow: A Case Report and Review of Literature

**DOI:** 10.7759/cureus.36449

**Published:** 2023-03-21

**Authors:** Ehab F Alsaygh, Waleed K Abduh, Alwaleed A Alshahir

**Affiliations:** 1 College of Medicine, Taibah University, Medina, SAU; 2 Department of Surgery, Orthopedic Section, King Fahad Hospital, Almadinah Almunawwarah, SAU; 3 College of Medicine, King Saud Bin Abdulaziz University for Health Sciences, Riyadh, SAU

**Keywords:** ulnar nerve, intraneural ganglionic cyst, intraneural ganglion, cysts, cubital tunnel syndrome

## Abstract

Cubital tunnel syndrome is a common disorder that affects the upper limb and involves compression of the ulnar nerve. However, this syndrome is rarely caused by multiple intraneural ganglion cysts. Of all intraneural ganglion cysts, only 9% affect the elbow. This study presents a case report of a 73-year-old female patient who manifested pain, numbness, tingling, and paralysis of the medial aspect of her left forearm, fourth, and fifth fingers of the left hand for six months. Intraoperative findings showed multiple intraneural cysts at the left elbow, which were confirmed via histopathology. The cysts were surgically excised, whereas the ulnar nerve was released into the cubital tunnel and anteriorly transposed. Complete sensory and motor recovery were achieved. Although similar cases of intraneural cysts were reported in the literature, this case has the uniqueness of the unusual number and site of intraneural cysts in the ulnar nerve on the background of osteoarthritic changes. Therefore, the aim of reporting this case is to increase awareness of the presence of these cysts when the symptoms are severe.

## Introduction

Following carpal tunnel syndrome, cubital tunnel syndrome is the most prevalent nerve compression neuropathy in the upper limb [1]. A known cause of this syndrome is the presence of a cyst that compresses the ulnar nerve. However, it is uncommon to observe these cysts when the occurrence is intraneural or more rarely when multiple cysts compress the intraneural of the ulnar nerve. Although these cysts are known in the lower extremities by affecting the common peroneal nerve, in the upper extremities, these cysts are rare with only 9% of them affecting the ulnar nerve at the elbow [2].

Patients usually present with compression symptoms such as pain, numbness, and loss of normal hand movements. Advanced stages of this syndrome lead to irreversible muscle atrophy and contracture of the hand (clawing). Symptoms can be relieved by ulnar nerve decompression, which prevents progression into advanced stages of dysfunction. Furthermore, it can be treated surgically using a variety of techniques [3]. the intraneural ganglionic cysts are rarely found to be causative of cubital tunnel syndrome. Based on our information, only three cases were reported previously with the presence of multiple intraneural cysts at elbow [1,4,5]. In our study, we aim to report a case of cubital tunnel syndrome caused by multiple intraneural ganglion cysts of the ulnar nerve at the elbow in view of an osteoarthritic joint.

## Case presentation

A 73-year-old female patient presented to the outpatient department complaining about pain, numbness, tingling, and paralysis in the medial aspect of her left forearm, fourth and fifth digits of the left hand. She had osteoarthritis of the elbow due to an old intercondylar distal humerus fracture 16 years ago, which was managed by nonoperative treatment. The pain started six months back, and was continuous, gradually increasing, and throbbing in nature. There were neither aggravating nor relieving factors. It affected her daily life activity greatly, and the condition was refractory to conservative treatments, which were in the form of nonsteroidal anti-inflammatory drugs (NSAIDS), rest with braces, and physiotherapy.

On examination, tenderness was observed in the left elbow at the medical aspect, the range of motion was 10-degree fixed flexion to 140-degree flexion, Tinel’s and Froment’s signs were positive. There were decreased sensations of the fourth and fifth fingers and a weak distal interphalangeal joint flexion of the fourth and fifth digits, however, no muscle atrophy of the first dorsal interosseous muscle. The compression of the ulnar nerve was later confirmed by a nerve conduction study. Computed tomography (CT) studies showed marginal, medial, and anterior osteophytes involving the humeroulnar joint with moderate to severe osteoarthritic changes. However, there was no obvious direct compression by the osteophyte at the cubital tunnel.

**Figure 1:**
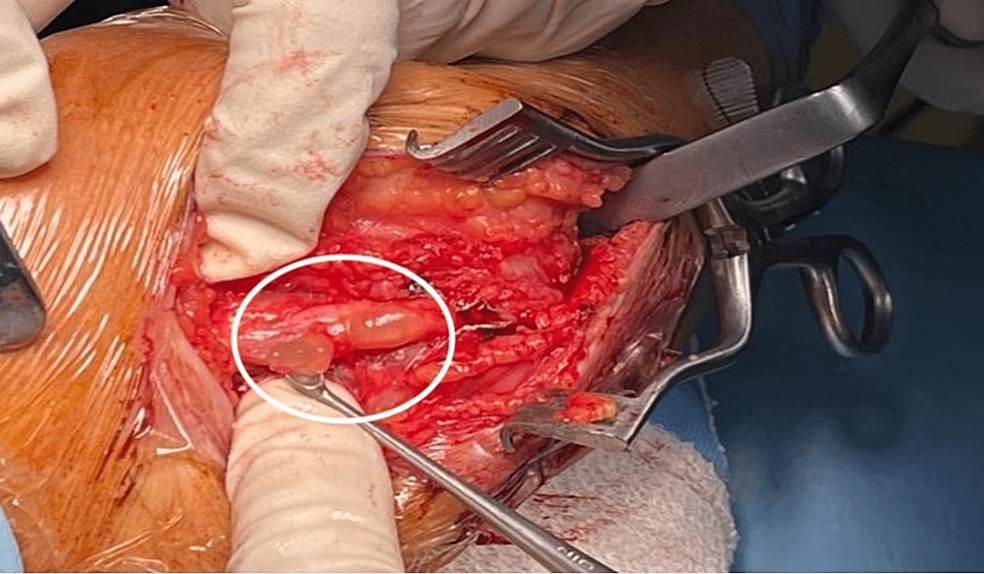
Multiple intraneural cysts were observed in the ulnar nerve

After explaining the procedure to the patient and obtaining consent, open surgery for release of the ulnar nerve was carried out. After incising 12 cm along the cubital tunnel, multiple intraneural cysts were observed in the ulnar nerve. Furthermore, no osteophyte compressing the nerve could be found at the tunnel (Figure 1).

**Figure 2:**
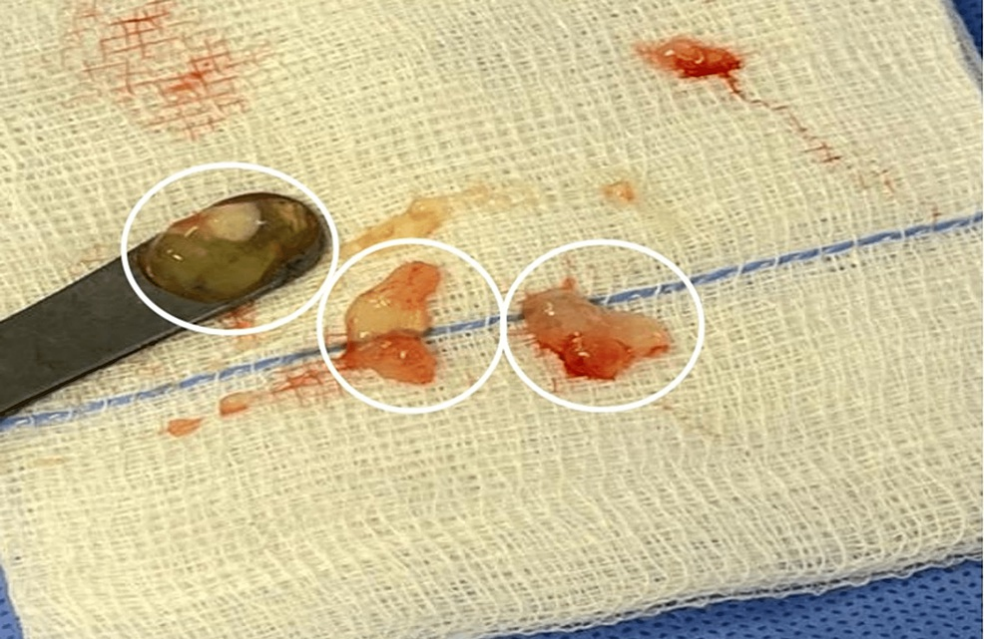
Cysts were found to be filled with mucinous fluid

The sides of compression that were released are the arcade of Struthers, intermuscular septum, Osbourne’s ligament, two heads of flexor carpi ulnaris, and deep flexor pronator aponeurosis. Then, the ulnar nerve sheath was incised, and a sample of the cysts was taken for histopathology which confirmed the diagnosis of multiple intraneural ganglionic cysts (Figure 2). Consequently, the ulnar nerve was released by anterior transposition. Following the transposition, the nerve was stable when tested with elbow flexion and extension and the wound was then closed with over drain. Lastly, our patient was followed up for six months and a complete recovery with no functional limitations was achieved.

## Discussion

Intraneural ganglionic cysts are rarely found in the ulnar nerve at the elbow. In their systematic review, Desy et al. found a prevalence of 9% of these cysts at the elbow. More rarely was the presence of multiple intraneural cysts of the ulnar nerve in the elbow, which to our knowledge, only three cases were presented in the literature [1,4,5].

The etiology of these cysts has been controversial. Yet, in the literature, a pattern of these cysts seems to develop in patients with a history of trauma or arthritic changes such as osteoarthritis, allowing the articular unification theory suggested by Spinner et al. to be a reasonable speculation behind these cysts [4,6-8]. Our patient similarly had history of distal humerus fracture and osteoarthritic changes of the elbow.

Although Chang et al. stated that intraneural cysts are more common in males, which is the case most commonly, our patient and Papadopoulos et al. on the contrary were elderly females [4,9].

The definitive diagnostic modality for such cysts is magnetic resonance imaging (MRI) or ultrasonography (US). Cubital tunnel syndrome is a common pathology, therefore, using such methods of diagnosis is neither routinely done nor cost-effective when the etiology is clear. However, the necessity for such methods could be justified when the etiology is vague. Thus, a diagnosis of multiple intraneural cysts was made by US along the ulnar nerve near the elbow in the case presented by Suparno et al. [5]. In this case, a CT scan and a nerve conduction study were performed preoperatively. Nevertheless, the diagnosis of multiple small cysts was achieved intra-operatively similarly to Li et al., and Papadopoulos et al. [1,4]. Moreover, in a study performed by Novac et al. 79% of the surgeons that participated recommended the use of a nerve conduction test or electromyography before undergoing surgery [10].

The excision of the articular branch and decompression of the swollen nerves is usually the treatment method of these intraneural ganglionic cysts [2,11]. The type of intervention is often chosen depending on the degree of nerve compression, the surgeon’s choice, and the unique circumstances of the patient [12]. Despite the fact that there are several therapies to select from, they do not all have the same effects.

In a review of the literature, Desy et al. discovered a higher risk of recurrence following initial surgery [11]. Hence, a proper assessment and therapy selection is critical. In the study presented by Novac et al., most surgeons choose subcutaneous anterior transposition as the primary operative treatment [10]. In addition, the recurrence rate reported by Desy et al. was 11% following primary surgery involving only resection of the cysts, and the mean time to recurrence was at 22 months. Moreover, the recurrence rate of 11% was among the lowest treatment options presented in their study, therefore, we believe that following up our patient for six months was sufficient in view of the surgical intervention that was used [11].

Table 1 summarizes all cases that were found in the literature with cubital tunnel syndrome caused by multiple intraneural cysts at elbow.

**Table 1:** Review of the literature on cubital tunnel syndrome caused by multiple intraneural cyst at elbow IGC: intraneural ganglionic cysts

Authors, years	study	Number of cases	Presence of osteoarthritic changes	Presenting signs and symptoms	Diagnostic tools of IGC	Result, follow-up period
Suparno et al., 2020 [5]	Case report	1	None	Numbness, tingling, clawing, and hypothenar wasting	US	Significant improvement, no recurrence on 3 - 24 month post-operatively
Li et al., 2012 [1]	Case report	1	present	Numbness, weakness, clawing, hypothenar and interosseous atrophy	Intra-operatively	Complete relief, no follow up
Papadopoulos et al., 2019 [4]	Case report	1	present	Pain, numbness, decreased sensation	Intra-operatively	Complete recovery, no recurrence on follow-up 3 month post-operatively
Present case, 2022	Case report	1	Present	Numbness, tingling, paralysis, decreased sensation	Intra-operatively	Complete recovery, no recurrence on follow-up 6 month post-operatively

In this case, multiple intraneural cysts were found upon exploration of the ulnar nerve, and there exist different treatment options. Decompression of the ulnar nerve is most commonly treated with medial epicondylectomy, simple decompression, or anterior transposition (subcutaneous, intramuscular, or submuscular) [10]. However, it is recommended to perform a simple decompression as the first option. This recommendation was due to the surgical simplicity, preservation of vascularization, and quick postoperative rehabilitation. Anterior transposition is suggested if subluxation is observed during surgery. In most cases, there is complete sensory and motor recovery [13].

## Conclusions

The etiology of cubital tunnel syndrome as multiple intraneural cysts compressing the ulnar nerve is rare. However, patients presenting with severe symptoms of ulnar compression and a history of osteoarthritis should raise the clinical suspicion of the presence of such an etiology. Finally, complete recovery could be achieved by anterior transposition and release of the ulnar nerve.
